# Effect of implementation of mental health services within primary care on GP detection and treatment of mental disorders in Israel

**DOI:** 10.1186/s13584-023-00553-0

**Published:** 2023-01-30

**Authors:** Neil Laufer, Nelly Zilber, Pablo Jeczmien, Royi Gilad, Shai Gur, Hanan Munitz

**Affiliations:** 1 Jaffa Mental Health Clinic, Ministry of Health, Tel Aviv-Yafo, Israel; 2 Falk Institute for Mental Health Studies, Kfar Shaul Mental Health Centre, Jerusalem, Israel; 3 Davidson Mental Health Clinic, Shalvata Mental Health Centre, Hod Hasharon, Israel; 4 Geha Psychiatric Hospital, Beilinson Campus, Petakh Tiqva, Israel; 5 Clalit Health Services, Tel Aviv-Yafo, Israel; 6 Herzliya Mental Health Clinic, Hadar Street 2, 46290 Herzliya, Israel

**Keywords:** Primary care, Mental health service models, GPs, Mental disorders, Detection, Treatment

## Abstract

**Background:**

Psychiatric morbidity is frequent in primary care, but a substantial proportion of these psychiatric problems appear to be neither recognized nor adequately treated by GPs. There exists a number of models of introduction of mental health services (MHS) into primary care, but little data are available on their effect on GPs’ detection or management of mental disorders. The study aimed to measure the effect of referring patients to a psychiatrist within primary care (Shifted OutPatient model—SOP) or consultation of psychiatrists by the GPs (Psychiatric Community Consultation Liaison—PCCL) on the detection and treatment of mental disorders by GPs.

**Methods:**

In six primary care clinics in Israel (three “SOP clinics” and three “PCCL clinics”), GP detection of mental disorders and treatment of GP-detected cases were evaluated before and after provision of 1-year MHS, according to GP questionnaires on a sample of primary care consecutive attenders whose psychological distress was determined according to the GHQ12 and psychiatric disorders according to the Composite International Diagnostic Interview.

**Results:**

After model implementation, a significant reduction in detection of mental disorders was found in SOP clinics, while no significant change was found in PCCL clinics. No significant change in detection of distress was found in any clinic. An increase in referrals to MHS for GP-diagnosed depression and anxiety cases, a reduction in GP counselling for GP-detected cases and those with diagnosed anxiety, an increased prescription of antidepressants and a reduced prescription of antipsychotics were found in SOP clinics. In PCCL clinics, no significant changes in GP management were observed except an increase in referral of GP-diagnosed depression cases to MHS.

**Conclusions:**

MHS models did not improve GP detection of mental disorders or distress, but possibly improved referral case mix. The SOP model might have a deskilling influence on GPs, resulting from less involvement in treatment, with decrease of detection and counselling. This should be taken into consideration when planning to increase referrals to a psychiatrist within primary care settings. Lack of positive effect of the PCCL model might be overcome by more intensive programs incorporating educational components.

## Introduction

Psychiatric morbidity is frequent in primary care, and general practitioners (GPs) are the main providers of care for most patients with common mental disorders [1–3]. Further, the Covid pandemic has resulted in increased psychosocial risk factors and associated psychiatric morbidity [4] and decreased access to psychiatric services, which might necessitate more treatment of mental disorders by GPs [5]. However, data in many countries, including Israel, have shown that a substantial proportion of these psychiatric problems appear to be neither recognized nor adequately treated by GPs [1, 6–8].

Several models of mental health services (MHS) within primary care have therefore been developed, which can be divided into replacement and collaborative type models [9]. The Shifted OutPatient (SOP) model consists of treatment of patients by the psychiatrist within the primary care clinic in order to increase access to psychiatric treatment and has been shown to increase treated prevalence of mental disorders [10]. Within the context of this replacement model, there is a possibility of consultation of the GPs with the psychiatrist but in practice it is generally limited. A common collaborative model is the Psychiatric Community Consultation Liaison model (PCCL), consisting of regular meetings of a psychiatrist with the primary care staff who remains the provider of mental health treatment [11]. Only a few studies, none of them in recent years, have assessed the effect of these models of MHS within primary clinics on GP detection and treatment of mental disorders [12, 13].

In the last 20 years “collaborative care” models have also been developed, where collaboration in treatment of patients with *GP diagnosed* mental disorders takes place between GPs and mental health professionals including psychiatrists. Analysis of these models primarily showed the symptomatic outcome of patients treated in these services, which has often been positive [14–16]. Only one time series study reported the effect of the program on GP diagnosis, as recorded in medical records, but without parallel objective assessment of prevalence at the time of the study [17].

In Israel, accessibility and use of primary health care services have been shown to be relatively high, with very high rates of primary care clinic visits relative to Europe and the US [18]. Primary care is provided by “Kupot Cholim” (health maintenance organizations—HMOs) within the framework of a compulsory health insurance, whereas MHS at the time of the study were funded and provided mainly by the state in public outpatient mental health facilities within the community or in psychiatric hospitals, with variability in availability and accessibility. Prior to the present study, replacement and collaborative type models had been implemented only sporadically and without any evaluation.

The aim of the present study was to implement the SOP and PCCL models in primary care clinics in Israel and to assess and compare their effect on GP detection and treatment of mental disorders among adult patients.

## Methods

### Intervention models

The SOP and PCCL models (“the models”) were randomly allocated to six primary health care centers in central Israel, where no mental health professionals had worked in the previous 5 years, 3 “SOP clinics” and 3 “PCCL clinics”. Four clinics served populations with low socioeconomic status and 2 with middle socioeconomic status. The models were implemented during the period 13/08/2001–05/10/2003 by a senior psychiatrist (one in each clinic) working in ambulatory MHS in the region. Before service implementation, the investigator clinicians met with the psychiatrist and the primary care team in each clinic, and uniform guidelines and policies were presented and discussed.

In the SOP model, the psychiatrist came every two weeks to the clinic for 4 to 5 h and assessed the patients referred by the GP and started treatment, with an aim to transfer patients’ care back to the GP after symptomatic improvement. The model was implemented for 1 year and, during the following 6 months, no new patients were accepted but the psychiatrist continued treatment when necessary. Consultation with the GP was minimized, and the main feedback of the psychiatrist was a written summary of assessment and treatment.

In contrast, the planned PCCL model consisted of a meeting of the psychiatrist with the primary care team once a month and with each GP separately every two weeks in one clinic and once per month in the other 2 clinics as decided by the clinics. Patients could also be jointly assessed by their GP and the psychiatrist, during the team meetings or during patients’ visit to their GP, when indicated. The model was implemented for one and a half year, except for one clinic (with only 2 GPs) where the program was implemented for only 1 year.

In both models, patients needing multidisciplinary or urgent treatment continued to be referred by the GPs to existing MHS.

### Process and instruments

At each clinic, before and after implementation of the program, consecutive patients, aged 18 to 65 years, waiting to see their GP were asked to provide written informed consent and fill in the General Health Questionnaire (GHQ12), a screening instrument for psychological distress [19]. The study was approved by the ethical committee of Geha Mental Health Centre. In order to focus on the effects of model implementation on the detection and treatment of common mental disorders, patients with schizophrenia/other psychotic illness, moderate to severe dementia, mental retardation and severe communication difficulties were excluded. A total of 2720 were eligible and 95.7% of them completed the GHQ-12 questionnaire (N = 2603). Data from the patients of 2 GPs who stopped working in the clinic during the study were not included in the analyses as well as patients for whom the GP did not fill in the form about their psychological morbidity and treatment, so that the analysis of detection and treatment of mental disorders by the GPs was finally done on 2347 patients—1252 during the year preceding the provision of new mental health services in the clinics (571 in SOP clinics and 681 in PCCL clinics) and 1095 after at least 1 year of model implementation (523 in SOP clinics and 572 in PCCL clinics)—all patients in the SOP clinics were sampled during the 6 months following the 1-year implementation of the program; in the PCCL clinics, most patients were also sampled after the end of the program. Patients treated within the model services were excluded from the post-model consecutive patient sample.

Psychiatric diagnoses were assessed on a stratified random sample of the patients based on GHQ12 scores where a high score was defined as one within the 80th–100th percentiles, a median score one within the 60th–80th percentiles, and a low score one within the 1st–60th percentiles. A stratified random sample was then drawn from the three GHQ strata: 100% of the patients with high scores, 35% of those with medium scores, and 10% of those with low score (details in [1, 2]). Diagnostic assessment providing ICD-10 diagnoses included the primary care version of the CIDI (Composite International Diagnostic Interview) [1], the Alcohol Use Disorders Identification Test [20], and three modules added from the CIDI 2.1 version [posttraumatic stress disorder (PTSD), obsessive compulsive disorder (OCD) and social phobia (SP)] [21]. All diagnostic instruments will be referred to as “CIDI”. Interview completion rate was 61.2%; 302 patients before and 199 after model implementation were interviewed.

In each sample no statistically significant differences between GHQ 12 and CIDI completers and refusers were observed in demographic characteristics (gender, age) and psychological pathology according to GP assessment. The study samples can therefore be considered as representative of consecutive patients in the clinics.

For each eligible patient who was sampled, the treating GP completed an Encounter Form that included assessment of the overall severity of the patient’s psychiatric morbidity in the past 12 months on a 5-point severity scale [completely healthy (0), subclinical symptoms (1), mildly ill (2), moderately ill (3) and severely ill (4)], with a score of 2 or more indicating detected mental health disorder (noted as GP^+^), while non-case will be noted as GP^−^. For GP^+^ patients, the GP would classify the disorder into diagnosis or symptom categories based on ICD10 Primary health care chapter headings such as depression, anxiety or dissociative/conversion symptoms.

#### GP detection and treatment of psychiatric disorders

The CIDI case definition included all threshold diagnoses assessed. Depressive disorders category included current depressive episode, recurrent depressive disorder and dysthymia. Anxiety disorders included general anxiety disorder, panic disorder, agoraphobia, PTSD, OCD and SP. Somatization disorders included somatization disorder, hypochondriasis, and neurasthenia. Somatoform pain disorder was not included due to issues of validity.

The rate of GP detection by the GPs of the patients’ mental health problems was defined as the percentage of patients detected by the GPs among patients having a psychiatric diagnosis according to the CIDI or among patients positive according to the GHQ-12—a score of 4 or above, as in a number of other studies, indicating a probable case or psychological distress; the use of the GHQ increased the power of the analysis because, as described above, many more patients filled the GHQ than the CIDI. The accuracy of GPs’ diagnosis, first defined as the percent of GP diagnosed patients with a given diagnosis among patients suffering from this diagnosis according to the CIDI was poor. GPs often diagnose mental disorder without specification and, while they indicated only one diagnosis, the comorbidity between diagnostic groups according to the CIDI was very high in this sample [2]. We therefore only examined GP detection of any disorder (GP^+^), separately among patients with depressive disorders, anxiety disorders and somatization disorders according to the CIDI.

When a patient was considered by the GP as a case, the treatment given was indicated on the Encounter Form. Treatment was classified as non-pharmacological or pharmacological. The former included: no non-pharmacological treatment; general psychosocial intervention (giving practical/social help, referral to nurse or social worker); referral to mental health professional (mostly MHS); counseling (discussing problems or giving advice); other (included further physical investigation and other non-psychosocial interventions). Psychopharmacological treatment categories included: no psychopharmacological treatment, sedatives (anxiolytics, tranquilizers, hypnotics), antidepressants (tricyclic antidepressants, selective serotonin reuptake inhibitors, selective noradrenaline reuptake inhibitors) and antipsychotic drugs, as well as other treatments (non-psychotropic drugs, such as vitamins, herbs or analgesics).

Treatments were analyzed for GP^+^ cases and GP defined depressive cases, anxiety cases and cases with different categories of somatoform symptoms and disorders.

## Results

Twenty-two GPs participated in the study, half in each model, 12 females and 10 males, including 12 specialists in Family Medicine. The mean age of the GPs was 49.2 (S.D. 8.8). The mean number of years since medical qualification was 23.5 (S.D. 8.4).

### Model implementation

The implementation of the SOP model was comprehensive, with all clinic sessions taking place as planned. Not all invited patients, however, showed up: they came to three quarters of their first appointment with the psychiatrist. The psychiatrists examined 195 patients, for whom there were 94 follow-up appointments, i.e. at least 51.8% of the 195 patients who came were seen only once for evaluation and/or treatment recommendation during model implementation and then the GP continued the patient’s follow-up. For those who were seen for follow-up appointments, the compliance of the patients for these visits was higher, with 82.5% attendance at appointments.

In the PCCL clinics, out of 34 potential team meetings, 21 took place (61.8%) and out of 36 potential meetings with individual GPs, 27 (75%) took place. Adjusting for number of GPs in the clinics (the 3 clinics had respectively 2, 3 and 6 GPs and were weighted 0.18, 0.27 and 0.55 respectively), 60% of team meetings and 83% of individual meetings took place. The participation rate of the different GPs in the meetings ranged between 69 and 100%, with a mean of 92%. In the team and individual sessions, 86 cases were presented for consultation with the psychiatrist, of whom19 (22%) were examined by both psychiatrist and GP, when joint examination was considered useful for diagnosis and/or treatment.

### Effect of model implementation on GP detection of mental health problems

Both before and after implementation of the models, there was a significant correlation between GP detection of a mental health disorder and case definition according to the CIDI (p = 0.0001 and p = 0.002 respectively). The detection rate before implementation of the models was not significantly different in SOP (41.9%) and PCCL clinics (42.1%). After model implementation, in the SOP clinics there was a significant decrease in GP detection rate (from 41.9 to 19.6%, p = 0.002), and the correlation between GP detection and caseness according to the CIDI was no longer significant. In the PCCL clinics, no significant change in detection rate was observed after model implementation (Table 1).

**Table 1:** Number (%) of mental disorder cases detected by GPs: caseness according to GHQ-12 (n = 2347) and CIDI (n = 458); comparison of GP detection before and after-implementation of the SOP and PCCL models

Model	Caseness according to GHQ				Caseness according to CIDI			
	Non-case		Case		Non-case		Case	
	Pre-model	Post-model	Pre-model	Post-model	Pre-model	Post-model	Pre-model	Post-model
*SOP*								
GP Non-case	396 (92.3)	387 (90.8)	119 (69.2)	75 (77.3)	51 (86.4)	34(87.2)	43 (58.1)	37 (80.4)
GP Case	33 (7.7)	39 (9.2)	53 (31.8)	22 (22.7)^1^	8 (13.6)	5 (12.8)	31 (41.9)	9 (19.6)^2^
Total	429 (100)	426 (100)	172 (100)	97 (100)	59 (100)	39 (100)	74 (100)	46 (100)
*PCCL*								
GP Non-case	415 (87.4)	411 (86.7)	138 (67.0)	65 (77.3)	51 (92.7)	36 (94.7)	55 (67.9)	34 (65.4)
GP Case	60 (12.6)	63 (13.3)	68 (33.0)	33(33.7)^3^	4 (7.3)	2 (5.3)	40 (42.1)	18 (34.6)^4^
Total	475 (100)	474 (100)	206 (100)	98 (100)	55 (100)	38 (100)	95 (100)	52 (100)

Comparison of case detection pre and post model: ^1^ *p* = 0.153; ^2^ *p* = 0.012; ^3^ *p* = 0.908; ^4^ *p* = 0.374

Comparison of GP detection of a mental health disorder and presence of psychological distress according to the GHQ 12 showed a significant correlation between the two (p < 0.01 both before and after model implementation). The detection rate before implementation of the models was not significantly different in SOP clinics (31.8%) and PCCL clinics (33.0%). After model implementation, there was a non-significant reduction of detection for SOP clinics (from 31.8 to 22.7%) and no change for PCCL clinics (from 33.0 to 33.7%)—Table 1.

When analysis was done among patients with specific diagnostic categories according to the CIDI, in SOP clinics a statistically significant reduction in detection of depressive disorders (51.5–23.5% p = 0.004) and of anxiety disorders (50–5.3% p = 0.0006) and no significant change in detection of somatization were found, with no significant change in PCCL clinics (Table 2).

### Effect of model implementation on GP treatment of mental health problems

Before service implementation, the distribution of both non-pharmacological and pharmacological treatments of GP^+^ cases was similar in both sets of clinics. For GP^+^ cases after model implementation, no change was observed for general psychosocial interventions; however, there was a near significant increase in the percentage of patients referred to a mental health professional (MHP) in the PCCL clinics (from 16.3 to 27.1% of cases, p = 0.051) and a significant decrease in GP counseling rate in the SOP clinics (from 23 to 6.3% of cases, p = 0.007). An increase in the use of antidepressants (from 21.8 to 42.2% of cases, p = 0.007) and a reduction in use of antipsychotics (from 9.2 to 0% of cases, p = 0.034) was found in the SOP clinics (Table 3).

**Table 2:** Number (%) of mental disorder cases detected by GPs among cases with depression, anxiety or somatization disorders according to CIDI (n = 458); comparison of GP detection before and after-implementation of the SOP and PCCL models

Model	CIDI depression case		CIDI anxiety case		CIDI somatization case	
	Pre-model	Post-model	Pre-model	Post-model	Pre-model	Post-model
*SOP*						
GP Non-case	50 (48.5)	26 (76.5)	27 (50.0)	18 (94.7)	15 (60.0)	8 (66.7)
GP Case	53 (51.5)	8 (23.5)^1^	27 (50.0)	1(5.3)^2^	10(40.0)	4 (33.3)^3^
Total	103 (100)	34 (100)	54 (100)	19(100)	25 (100)	12 (100)
*PCCL*						
GP Non-case	30 (48.4)	25 (65.8)	34(51.5)	17 (65.4)	20 (42.6)	7 (50.0)
GP Case	32 (51.6)	13 (34.2)^4^	32 (48.5)	11 (34.6)^5^	27 (57.4)	7 (50.0)^6^
Total	62(100)	38 (100)	66 (100)	28 (100)	47 (100)	14 (100)

Comparison of case detection pre and post model: ^1^ *p* = 0.004; ^2^ *p* = 0.0006; ^3^ *p* = 0.695; ^4^ *p* = 0.090; ^5^ *p* = 0.413; ^6^ *p* = 0.622.

**Table 3:** Number (%) of mental disorder cases given nonpharmacological or pharmacological treatments by GP’s, among GP-diagnosed cases, GP-diagnosed depression and GP-diagnosed anxiety; comparison of GP treatment before and after-implementation of the SOP and PCCL models

	GP diagnosed cases				GP diagnosed depression				GP diagnosed anxiety			
	SOP		PCCL		SOP		PCCL		SOP		PCCL	
	Pre-model	Post-model	Pre-model	Post-model	Pre-model	Post-model	Pre-model	Post-model	Pre-model	Post-model	Pre-model	Post-model
*Non-pharmacological interventions*												
No non-pharmacological treatment	40 (42.6)	31 (49.2)	66 (53.7)	38 (39.6)^1^	7 (41.2)	0 (0.0)^4^	21 (80.8)	13 (41.9)^5^	16(42.1)	11 (45.8)	27 (79.4)	12 (52.2)
General psychosocial interventions^a^	8 (8.5)	9 (14.3)	7 (5.7)	14 (14.6)	2 (11.8))	1 (5.3)	0(0.0)	1 (3.2)	3 (7.9)	1 (4.2)	3 (8.8)	6 (26.1)
Referral to Mental Health Professional	18 (19.1)	18 (28.6)	20 (16.3)	26 (27.1)^2^	4 (23.5)	15 (78.9) ^6^	2 (7.7)	15 (48.4)^7^	5 (13.2)	9 (37.5)^8^	2 (5.9)	3 (13.0)
Counseling^b^	21 (22.3)	4 (6.3) ^3^	19 (15.4)	8 (8.3)	3(17.6)	2(10.5)	3(11.5)	2(6.5)	13(34.2)	2 (8.3)^9^	2 (5.9)	2 (8.7)
Other^c^	7 (7.4)	1 (1.6)	11 (8.9)	10 (10.4)	1(5.9)	1(5.3)	0 (0.0)	0 (0.0)	1(2.6)	1(4.2)	0 (0.0)	0 (0.0)
Total	94 (100)	63 (100)	123 (100)	96 (100)	17 (100)	19 (100)	26 (100)	31 (100)	38 (100)	24 (100)	34 (100)	23 (100)
*Pharmacological treatments*												
No drug treatment	31 (35.6)	21 (32.8)	55 (44.7)	38 (42.2)	4 (23.5)	4 (21.1)	5 (19.2)	8 (25.8)	11 (28.9)	11 (45.8)	19 (55.9)	13 (56.5)
Anxiolytics, tranquilizers hypnotics	22 (25.3)	12 (18.8)	17 (13.8)	12 (13.3)	0 (0.0)	2 (10.5)	4 (15.4)	4 (12.9)	14 (36.8)	6 (25.0)	6 (17.6)	5 (21.7)
Antidepressant	19 (21.8)	27 (42.2)^10^	26 (21.1)	28 (31.1)	11 (64.7)	12 (63.2)	11 (42.3)	15 (48.4)	5 (13.2)	6 (25.0)	5 (14.7)	2 (8.7)
Antipsychotic	8 (9.2)	0 (0.0)^11^	9 (7.3)	7 (7.8)	2 (11.8)	0 (0.0)	3 (11.5)	3 (9.7)	3 (7.9)	0 (0.0)	3 (8.8)	1 (4.3)
Other^d^	7 (8.0)	4(6.2)	16 (13.0)	5 (5.6)	0 (0.0)	1 (5.3)	3 (11.5)	1 (3.2)	5 (13.2)	1 (4.2)	1 (2.9)	2 (8.7)
Total	87 (100)	64 (100)	123 (100)	90 (100)	17 (100)	19 (100)	26 (100)	31 (100)	38 (100)	24 (100)	34 (100)	23 (100)

^a^Giving practical/social help, referral to primary care nurse

^b^Discussing problems, advice

^c^Further physical investigation and other non-pharmacological interventions

^d^Non psychotropic medication –vitamins, herbal, analgesics

Significant differences in GP treatment pre and post model: ^1^ *p* = 0.038; ^2^ *p* = 0.051; ^3^ *p* = 0.007; ^4^ *p* = 0.007 (Yates correction); ^5^ *p* = 0.003; ^6^ *p* = 0 0.001; ^7^ *p* = 0.001; ^8^ *p* = 0.019; ^9^ *p* = 0.025; ^10^ *p* = 0.007; ^11^ *p* = 0.034(Yates correction)

For GP-diagnosed depressive disorders, a significant increase (p = 0.001) in referral to MHP was observed in both sets of clinics after implementation of the models. When these cases were removed from GP^+^ cases, there was a reduction in referral rate although not significant (SOP 18.2–6.8%, p = 0.08 and PCCL 18.5–16.9%, p = 0.79). For GP-diagnosed anxiety disorders, a significant increase in MHP referral (from 23.5 to 39.1%, p = 0.019) and a reduction in GP counseling (from 17.6 to 8.6% p = 0.025) was observed in SOP clinics.

No change in psychopharmacological treatments was shown for either model for GP diagnosed depressive or anxiety disorders. It is noteworthy that antidepressant treatment rates were already relatively high for depression before model implementation − 64.7% in SOP clinics and 42.3% in PCCL clinics (Table 3). There were too few cases to analyze GP diagnosed somatization disorders as they were diagnosed less frequently by the GPs.

## Discussion

### Effect of model implementation on GP detection of mental health problems

After implementation of the programs during at least 1 year, there was no improvement in the detection by the GPs of psychiatric disorder or distress (as determined respectively by the CIDI and the GHQ-12). In the SOP clinics, there was even a significant decrease in the detection by the GPs of mental disorders.

The difference in the results for the two types of models could be explained by the fact that in the PCCL clinics the GPs discussed patient’s problems with the psychiatrists while in the SOP clinics, the major aim of introducing psychiatrists into the clinics was to increase the accessibility of primary care patients to psychiatric care; contact was minimal between the GPs and the psychiatrists who only provided summary of diagnosis and treatment for referred cases. Thus, the GP had limited opportunity to learn from the psychiatrist and improve detection.

The significant decrease in detection of mental disorders after implementation of the program in SOP clinics was unexpected, although similar negative results were found in a study in Manchester [12] assessing a 3-year service where10 GPs could discuss patients’ problems with a community-based psychiatric team or with mental health professionals in primary care team meetings (similarly to our PCCL model) or could refer patients to weekly psychiatric clinics in the general practice (similarly to our SOP model). Comparing GPs of this study group with matched GPs using traditional hospital MHS, for both groups of GPs the study found a reduction in their ability to detect symptomatic psychiatric morbidity (defined according to GHQ28) over the study period, but this decrease only reached significance for practitioners without access to the new service. As detection by GPs of patients with psychological distress is affected by time limitations on the visits, the authors suggested that a new contract for GPs introduced during the study period might have played a role in the decrease in psychiatric morbidity recognition. In our study where no change in policy occurred, lack of improvement in detection even in the PCCL clinics, as found also in several studies where educational programs were introduced in primary care clinics, might thus be related to limited time for visits, which has been shown to be a crucial factor in detection of mental health problems [22, 23].

It is also possible that GPs invested less effort filling in questionnaires after the end of model implementation, but the lack of change in the percentage of false positives in our study, which was relatively low before and after implementation of the models (circa 10%), might not support this thesis. In our study, in the PCCL clinics there was no change in the detection rate whether the gold standard was CIDI or GHQ. In the Manchester study, there was also no change, while a significant decrease was observed in the control group, which indicates a beneficial effect of the implementation of these services. It might be the same in our study; unfortunately, we did not have control clinics where no model was implemented. Our results contrast a study in Sweden [13] that showed a significant increase in detection of anxiety and depression after 1-year implementation of a program that combined PCCL (with meetings every 2 weeks reviewing 5–6 patients with each GP) and 2–3 training sessions during the year, which consisted of lectures on various psychiatric disorders found in primary care. Thus, this program was more intensive and included an education component. A deficiency of our study might be that in the PCCL clinics not all potential meetings took place and there was no requirement of a minimum number of patients to be discussed and /or examined during the meetings. It is of note that the results of many studies of educational and training interventions to improve detection and treatment of mental health problems by GPs have been inconsistent [18]. Some studies on training interventions have shown no change in GPs’ detection of depression [24, 25] while others showed a reduction [26] and others an improvement [27, 28]. In the studies that showed improvement, the training was done in small groups and was very time-consuming. The training should also aim to increase level of commitment, motivation and interest in mental health, which have been suggested as relevant factors [29]. Further knowledge and interviewing skills, which are important for detection, might be difficult to impact with less intensive programs such as the models in our study.

In primary care, many patients are symptomatic, presenting significant symptoms of distress but not meeting definition of psychiatric disorders and a high comorbidity between disorders and symptoms in different symptomatic categories has been observed [1, 30, 31]. Studies indicate that the constructs of mental distress/disorders by GPs are different from those of the psychiatrists; for instance, a study on assessment of psychological distress by GPs showed their construct focused on a small proportion of the GHQ 28 symptoms and on different factors such as impairment, not included in the GHQ [32]. Further, specific diagnostic systems for GPs, including more dimensional approaches based on differences in clinical presentation of patients in primary care, have also been suggested as more clinically relevant [33, 34]. A possible explanation of the decrease in detection of categorical diagnoses in the SOP clinics is that anticipating working with psychiatrists might have encouraged the GPs to look more attentively for categorical diagnoses when filling the questionnaires. After the end of the program, the GPs might have again focused more on symptoms and other factors, whereas in PCCL clinics the consultation with a psychiatrist might have led to maintain focus on categorical diagnoses. Furthermore, the lack of reduction in detection of somatization disorders or distress after implementation of the models in both sets of clinics, in contrast to the reduction in detection of any disorder, of depressive and of anxiety disorders in the SOP clinics, could be explained by the greater overlap of GP constructs, mainly symptom based and of the criteria for somatization disorders or for distress according to the GHQ and by the better ability of GPs to identify distress than categorical diagnoses such as depression [35]. One of the strengths of our study is that, while most other studies used only GHQ as an indicator of mental disorder, our study also used a diagnostic instrument.

### Model effect on GPs referrals

In our study, after implementation of each model, there was a significant increase in referrals to MHS outside the primary care clinics in both types of clinics for GP-diagnosed depressive disorders and, in SOP clinics, also for GP-diagnosed anxiety disorders, which was responsible for the tendency to the increase in referrals to MHS for GP-diagnosed mental disorders (significant in the PCCL clinics). In the few studies which tested whether implementation of the models changed the referral rate, an increase in referrals was also observed, but the measure was done during the implementation and “referral” included also referral to the primary care MHS [36, 37]. These studies cannot thus be compared with ours where measures were done after service was stopped in the large majority of cases and changes would more likely reflect change in GPs’ referral preference or skills. In a study in Australia [38], where a PCCL type model was implemented, referral rates (not including referral to psychiatrists within the primary care clinics) were estimated after 18 months of service implementation (it is not clear whether during the implementation or after it ended). No increase in referral was reported: there was no significant change in referral to public services and a reduction of referral to private psychiatric treatment. In this study however, referral rates to different services were an estimate of the GPs and not rates based on questions about each patient. Since in general more severe cases are treated in hospital and less severe cases in private clinics, the decrease in referral to private clinics may indicate that the GPs felt confident enough to treat these cases by themselves and indeed they reported improvement in their skills. In our study on the contrary there was an increase in referrals of GP-defined depressive cases. Although increase in the number of referrals has been seen as evidence of an improved service with more people gaining access to MHS [39, 40], the case mix of referrals and appropriateness of referral has been viewed as more important [9, 41]. The selective increased referral of patients with depressive disorders for both models in our study might reflect referral of patients with more severe depression and thus improved service quality.

### Effect of model implementation on GP interventions

1. *Non-pharmacological intervention.* In SOP clinics, less counselling by the GPs was given to GP^+^ patients, and in particular GP-diagnosed anxiety disorders. Similar findings were described in the Manchester study [12] where the authors suggested a deskilling effect, when the replacement type MHS serves as a referral locus for patient treatment, replacing the GP as the therapist and reducing GPs’ involvement in counselling and psychological interventions. The increased referral to MHS of patients with anxiety in the SOP model might also indicate reduction of GP involvement in treatment. The negative effect on mental disorder detection in SOP clinics contrasting no significant reduction in PCCL clinicsmight indicate a deskilling effect by reducing GPs involvement in diagnostic assessment. This would also explain the contrast with PCCL clinics where the GPs continued treatment and acquired diagnostic skill through discussion with the psychiatrist. Our study supports past WHO recommendations [42] not favoring replacement models of primary care MHS, due to possible GP deskilling over time. Policies for increasing independent psychiatric services in the community including in primary care clinics, although increasing access to psychiatric treatment, might have a negative deskilling effect on GPs’ involvement in treatment.
2. *Pharmacological intervention*. In our study, for GP-detected mental disorders, an increase in the use of antidepressants was observed (statistically significant for SOP clinics), which is a positive effect of the implementation of the models, in view of the positive responses to antidepressants of patients with anxiety and depressive disorders. This was true even if the GP did not specifically define the disorder as depression or anxiety, as cases in this sample had mainly depressive or anxiety disorders [2]. Similar results were observed in the time series study [17] that showed increased prescription of antidepressants in medical records during the time of mental health collaborative care. Among GP-diagnosed depressive disorder patients, there was no increase in the already relatively high use of antidepressants before program implementation compared to rates reported in other centers [1]. After the program, severe cases might have been referred rather than prescribed treatment, possibly explaining the lack of change in anti-depressive drug treatment.

The reduction in the use of antipsychotics in the SOP model could be considered a positive effect on GP treatment in view of the lack of evidence of efficacy of antipsychotics in common non-psychotic mental disorders at the time of the study. This, as well as the significant increase in the use of antidepressants only in the SOP clinics, may be explained by the fact that the main feedback received by the GPs was about medication prescribed, while in the PCCL clinics the discussion with GPs focused on a wider range of diagnostic and treatment issues, such as psychosocial factors and interventions.

### Limitations of the study

1. The main limitation is that the study relates to data which were collected long ago—it is part of a larger one whose data were mainly collected in 2001–2004 [2]. In view of its not encouraging results, we decided not to focus our attention on this part of the study. Later, the present analysis was nevertheless further carried out in view of the publication of other negative later studies [e.g. 22,26] and of the increasing number of mental health problems in primary care clinics due to COVID pandemics.

Since our study, changes have occurred in the Israeli health system, which might have made our results less relevant today. The main change was, in 2015, a reform of mental health services in Israel, which added mental health treatment to the mandatory basket of health services provided by the HMOs [43]. The HMOs were allotted extra budgets to either provide or buy MHS from existing providers such as government mental health clinics. The reform aimed to increase access and availability of MHS in the community, and some additional ambulatory services (predominantly by the HMOs) and hospital alternative settings have been developed and opened, and some HMOs have tried to increase the number of psychiatrists in the community. Unfortunately, currently there are no data available regarding an eventual change in the use of psychiatric ambulatory services after the reform.

Possible effects of the reform that might have changed our results include:

- If there was an increased availability of MHS in the community, it might have led to an increased ease for GPs to refer patients to MHS and thus to less involvement of GPs in managements of patients with mental disorders (as was apparently the case after implementation of the SOP model), so that the decrease in detection in the SOP model might be somewhat smaller. However, waiting lists for referral to psychiatric clinics are still high and therefore these new services would be expected to have less impact on GP involvement with mental health problems than in the SOP model where the referral to a psychiatrist was on site and thus easier.
- The reform could have stimulated the interest of GPs in mental health, and the PCCL program might be followed more attentively by the GPs with the results possibly improved, although increased GP interest in mental health has not been reported.

Over the years other changes may also have happened, in the working environment of GPs such as changes in patients’ expectations telehealth, particularly during the limitations due to the COVID pandemics. However, there is no report on possible influence on GP detection and treatment of mental disorders.

Also, detection and treatment by GPs might have changed over the years, although no study reports such a trend. However, the results of our study should remain relevant since they do not relate to detection and treatment rates per se but to the effect of primary care mental health services on these parameters.

It is thus difficult to see how the reform as well as eventual other changes over the years could have substantially changed the results of the study. On the contrary, having done the study before the reform is in fact an advantage since it allowed to study the effect of a new primary care MHS without the possible interference of still ongoing changes due to the reform [44].

2. The study lacked control clinics where no service was implemented as discussed above.
3. A more comprehensive assessment of participation and an assessment of motivation and interest of the GPs, particularly in the PPCL program, might have been important.

### Conclusion and policy implications

Our findings suggest that two commonly used paradigms of primary care mental health services, as implemented in the present study, do not show a clear positive effect on GP detection and a minimal effect on treatment of mental disorders. Availability of psychiatrists in primary care for referral (the SOP replacement referral type model) and possibly increased accessibility of psychiatric referral in the community, although providing a positive effect on treated prevalence by a psychiatrist, might however have a deskilling influence on GPs caused by less involvement in treatment, with a decrease of detection and counselling. Both models may improve referral case mix with referral of possibly more severe or treatment-resistant cases to MHS, although further studies are needed regarding this point.

Improvement of quality of mental health care provided by GPs, the main providers of care for patients with common mental disorders, should be an important aim for planners of both general and mental health care systems. To the best of our knowledge, although the reform should have facilitated implementation of MHS in primary care clinics since both are provided or funded by the same HMO, no service or program to increase detection and treatment of mental health problems within primary care have been described or published since implantation of the mental health reform. The present study should promote focus of health service providers and planners on optimizing detection and management of common mental disorders by GPs and stimulate initiation and study of new paradigms of MHS delivery to primary care clinics.

The detailed results of the present study should have implications regarding the MHS which should be provided by the HMOs. Should a SOP-type program or any other program with direct treatment by psychiatrists in primary care be implemented, it should include more interaction and discussion of cases between the GPs and the psychiatrist in order to avoid decrease of involvement of GPs in treatment of mental disorders. Should a PCCL-type program be implemented, it would need to be more intensive and longer, probably incorporating other elements such as education, encouragement of commitment and interest in mental health or inclusion of incentives.

## Data Availability

The datasets used and/or analysed during the current study are available from the corresponding author [N.L] on reasonable request.

## Abbreviations

GPs: General practitioners
SOP: Shifted OutPatient Model
PCCL: Psychiatric Community Consultation Liaison model
HMOs: Health maintenance organizations
GHQ: General Health Questionnaire
CIDI: Composite International Diagnostic Interview
PTSD: Posttraumatic stress disorder
OCD: Obsessive compulsive disorder
SP: Social phobia
GP^+^: GP detected mental health disorder
MHP: Mental health professional
